# Association of mass media exposure with combustible and smokeless tobacco use among Indian population: findings from a panel survey

**DOI:** 10.1186/s12889-021-12459-0

**Published:** 2022-01-18

**Authors:** Ronak Paul, Rashmi Rashmi, Shobhit Srivastava

**Affiliations:** International Institute for Population Sciences, Deonar East, Mumbai, Maharashtra 400088 India

**Keywords:** Mass media exposure, Smoking, Smokeless tobacco consumption, Health behaviour, India Human Development Survey

## Abstract

**Background:**

Despite introducing different policies and initiatives, India is recognized as one of the global players in the tobacco epidemic race. Our study explores the association between tobacco consumption and mass media exposure among the Indian population, considering the contextual factors affecting the clustering at the community and state levels.

**Methods:**

Using two waves of the India Human Development Survey (IHDS) conducted in 2005 and 2012 for 16,661 individuals, the present study explores the association of mass media exposure and tobacco consumption in the short-term and the long-term period of Cigarettes and Other Tobacco Products Act (COTPA) implementation, which came into existence in 2004. Bivariate analysis using the chi-square test for association showed the correlation of tobacco consumption with its respective predictors. Multivariable analysis using three-level random intercept logit models showed the adjusted association between tobacco consumption and its relevant covariates and the extent of clustering of tobacco consumption behaviour of persons in the communities and states.

**Results:**

We found that watching television (TV) [(OR:1.03; CI:0.92–1.15) in 2004–05 and (OR:0.99; CI:0.88–1.12) in 2011–12], listening radio [(OR: 0.99; CI:0.90–1.10) in 2004–05 and (OR:1.04; CI:0.94–1.15) in 2011–12] and reading newspaper [(OR:1.02; CI:0.91–1.15) in 2004–05 and (OR:0.96; CI:0.87–1.06) in 2011–12] did not have any significant effect on consumption of combustible tobacco. Similarly, no effect of mass media was found on smokeless tobacco consumption. Further, the clustering of combustible and smokeless tobacco usage was higher at the community level than at the state level. In both rounds, smokeless tobacco consumption was found to be higher than combustible tobacco.

**Discussion:**

The present study provides evidence that COTPA has achieved its aim of nullifying the significant effect of mass media on combustible and smokeless tobacco consumption among the Indian population. However, the influence of state- and community- level clustering had failed in curbing the increment of smokeless tobacco consumption. There is a need for policy reforms to curb the significant impact of factors that promotes smokeless tobacco consumption in India. Further, initiatives must focus on specific communities from high-risk states, reducing the time and cost required for implementation.

## Background

Combustible and smokeless tobacco consumption is the most significant preventable cause of death worldwide [1]. Being the second-largest tobacco consumer, India is one of the global players in the tobacco epidemic race [2]. In 2016, tobacco consumption (including smoking, smokeless tobacco and second-hand smoke) had alone contributed 6% DALYs (Disability-adjusted life-years) in India, and the burden was higher among the men (8.3% of total DALYs) than in women (3% of total DALYs) [3]. Existing evidence from India has shown the trends in the prevalence of different forms of tobacco consumption [4–6]. Several studies linked combustible and smokeless tobacco consumption with family, friends, and peer influence (social support for tobacco use) [7]. A couple of studies have linked tobacco consumption with illiteracy and working stress [8, 9]. Other studies identified affordability and social acceptability [10], socioeconomic inequality [11], pro-tobacco media campaigns by tobacco companies to attract the population, especially the youngsters [12], as factors leading to a hike in smokeless and combustible tobacco consumption. Additionally, the powerful influence of films and advertisements featuring the macho image of characters who smoke has an everlasting impact on children and adolescents’ minds, leading them to adopt similar tobacco consumption behaviour [13]. Some studies have also shown the differential in smoking and smokeless tobacco usage across the Indian states and communities [5, 6]. A cross-sectional study from India had tried to reveal geographic variation in tobacco consumption and showed the importance of local contextual factors and policies that shape tobacco use [14].

Despite such prominent explanatory factors of tobacco consumption, a recent reduction in combustible tobacco has been noticed among Indian individuals [15, 16]. However, a different concern of increased smokeless tobacco consumption compared to smoking has come up [17]. It is worth noting that India, Bangladesh and Myanmar jointly contribute 71% of world smokeless tobacco consumers [18]. One study showed that a ban on smoking in public places in India had resulted in an increment in smokeless tobacco consumption [19]. Another research from three countries (Bangladesh, India, Nepal) of Southeast Asian regions revealed that tobacco companies’ misleading tobacco advertisements continuously drove smokers to the alternative of smokeless tobacco consumption [17]. The study noted that the marketing of tobacco products was done by promoting them as an inalienable part of the consumer’s lifestyle. Direct and surrogate advertising of these products through the media influences the consumers and encourages them to use them [20]. However, the Indian parliament had introduced the Cigarettes and Other Tobacco Products Act (COTPA), 2003, which came into effect from May 2004 to ensure that Indian people do not indulge in or increase their tobacco consumption by being influenced by the media content [21]. Ample evidence from India, since 2005, revealed that media exposure still plays an essential role in increasing combustible and smokeless tobacco usage among people [22, 23].

The present study examines the association between tobacco use and mass media exposure among the Indian population, considering the contextual factors that may affect the clustering of tobacco consumption at both community and state levels. The rationale for such analysis is as follows. First, minimal attempts have been made to understand the effect of the tobacco advertisement ban through mass media on the likelihood of combustible and smokeless tobacco use in the Indian context. One study using National Family Health Survey 2005–06 data (i.e., after COTPA act implementation) showed the association between smoking and smokeless tobacco use and mass media exposure among Indian adults aged 15–54 years [24]. However, it could not provide similar evidence for adolescents and the elderly. Second, rather than proceeding with a before-after impact assessment of the COTPA act, this study wants to utilize the panel nature of the India Human Development Survey (IHDS) dataset conducted in 2005 and 2012, to explore the association of mass media exposure and different tobacco consumption in the short-term (in the early phase of a 1-year leap) and the long-term (in the later phase of 7 years leap) period among the same individuals after the COTPA act implementation. Third, a dearth of evidence on the association of tobacco consumption and mass media exposure after considering the contextual level factors convince us to explore the variation in combustible and smokeless tobacco consumption clustering using a three-level multilevel approach.

## Methods

### Data source

This study used the India Human Development Survey (IHDS) round-I and round-II. IHDS round-I is a large-scale, nationally representative and multi-topic survey of 41,554 households across 382 districts of India conducted during 2004–05 [25]. The IHDS round-II, conducted during 2011–12, surveyed 42,152 households across 384 districts of India [26]. IHDS round-II re-interviewed 83% of the original households from round-I. National Council for Applied Economic Research (NCAER) India, in collaboration with the University of Maryland, USA, conducted both rounds of IHDS in all the Indian states and union territories (except for Andaman & Nicobar Islands and Lakshadweep). IHDS used multi-stage stratified random sampling, and further details regarding the sample selection procedure are available elsewhere [27, 28].

IHDS collected combustible and smokeless tobacco consumption data from 33,116 and 34,090 persons during round-I and round-II. The current study used the panel data of 16,661 individuals nested within 2175 communities across 33 states from both rounds for analysis. By community, this study refers to the primary sampling units (PSU), which are villages in rural areas and census enumeration blocks in urban areas, respectively. In our study panel, 61% of 16,661 persons smoked tobacco during round-I, which decreased to 41% during round-II. Moreover, nearly 59% of persons consumed smokeless tobacco in both rounds of IHDS.

### Outcome variables

The two outcome variables of this study are the binary indicators denoting whether an individual consumed combustible tobacco and smokeless tobacco, respectively. During IHDS round-I and round-II, interviewers collected information about “whether an individual smokes cigarettes, bidis or hookah” (combustible tobacco products) and “whether an individual chews tobacco or gutkha” (smokeless tobacco products), respectively. Persons who consumed one or more combustible or smokeless tobacco products were coded as “Yes” and otherwise coded as “No”. Both the outcome variables had no records with missing information in both rounds of the panel data.

### Explanatory variables

The three binary indicators of mass media exposure – whether anyone in the household “watches television (TV)”, “listens to radio”, and “reads the newspaper” are the explanatory variables in both rounds of IHDS. During both survey rounds, interviewers asked a respondent from each household about how often do people in the family “listen to radio”, “read the newspaper”, and “watch TV”. Owing to skewed distribution, households in the “sometimes” and “daily” categories were coded as “yes”; otherwise, they were recoded to “no”. The three explanatory variables had no records with missing information in both rounds.

### Control variables

The current study identified several confounding factors associated with tobacco consumption and mass media exposure among individuals based on existing research. The individual-level characteristics were – age group (children and youth, adults, elderly), sex (male, female), level of education (no formal schooling, 1–5 years of schooling, 6–10 years of schooling, more than 10 years of schooling), current working status (not working, working) and current marital status (currently married, currently not married). The household characteristics were – wealth quintile (poorest, poor, middle, rich, richest), the caste of household head (Other Backward Classes (OBC), Scheduled Castes (SC), Scheduled Tribes (ST), others) and religion of household head (Hinduism, Islam, others). Further, we included the following contextual variables at the community level – Percentage of individuals in the community with no formal education (0 to 25%, 25 to 50%, 50 to 75%, 75 to 100%), Percentage of individuals in the community from poorest/poor wealth quintile (0 to 25%, 25 to 50%, 50 to 75%, 75 to 100%) and Percentage of individuals in community belonging to SC/ST caste (0 to 25%, 25 to 50%, 50 to 75%, 75 to 100%). Additionally, place of residence (rural, urban) is included as a community-level characteristic. Country regions (central, northern, southern, western, eastern and north-eastern) is included as a state-level characteristic.

In our study sample, the population distribution by age is skewed with fewer people in the young and old age categories. Therefore, persons aged 24 years and less were coded as “children and youth”, those aged between 25 to 64 years were coded as “adults”, and “elderly” included persons 65 years and above in the age group variable.

We estimated the wealth quintile for all households in both rounds of IHDS. We generated the wealth scores using the standard procedure of principal component analysis using household data on asset ownership, building material type, type of household water source, type of household sanitation facility and the number of living rooms in the household. Details of the standard procedure are available elsewhere [29, 30]. Based on the wealth score, the families were classified into five categories (poorest, poor, middle, rich, richest) such that the households with the lowest 20 percentile score belonged to the “poorest” category, families with the next low 20 percentile score belonged to the “poor” class and so forth [30, 31].

Contextual characteristics of the community where a person belongs are known to influence their behaviour. Therefore, in a multicultural country like India, these factors might significantly affect the tobacco consumption behaviour of individuals. Accordingly, we controlled for the effect of the community’s educational, economic, and social composition. The community-level education composition has been shown by the percentage of the population with no formal education in a community. The community social composition is determined by the percentage of Scheduled Tribes (ST) and Scheduled Castes (SC) population in a community. The percentage of the people belonging to the poorest and poor wealth quintile shows the economic composition of the community. All the three indicators have four categories – 0 to 25%, 25 to 50%, 50 to 75%, and 75 to 100%. We divided the 33 Indian states and union territories into six regions based on administrative classification [32].

### Statistical methods

We undertook bivariate and multivariable analyses to fulfil the study objectives. We performed two similar sets of evaluations separately for examining the association of combustible and smokeless tobacco consumption with mass media exposure among the same population during round-I and round-II, respectively. Bivariate analysis showed the correlation of tobacco consumption with its respective predictors, using the chi-square test for association. Multivariable analysis using three-level random intercept logit models showed the association between tobacco consumption and its relevant covariates and the extent of clustering of tobacco consumption behaviour of persons in the communities and states, respectively [33, 34]. We used a three-level multilevel model owing to the hierarchical structure of the data where 16,661 persons (level 1) are nested within 2175 communities (level 2), which in turn are nested within 21 states (level 3). Note that, owing to the skewed distribution of population across the 33 states, we have merged them into 21 groups such that the five union territories (Delhi, Chandigarh, Daman & Diu, Dadra & Nagar Haveli and Pondicherry), the seven north-eastern states (see section Control variables) and Maharashtra & Goa are in distinct groups. Further, a multilevel logit model was necessary, as the outcome variables of this study are binary.

The use of a three-level model allows us to adjust for unexplained inter-community and inter-state variation (heterogeneity) in the risk of tobacco consumption. These models give odds ratios that are the odds of tobacco consumption among all the persons in a particular category compared to the reference category for the specific explanatory variable, given that the effect of all the other explanatory variables and the group-level effects remains constant. The multivariable models give the Intra-class Correlation Coefficient (ICC) that measures the expected degree of similarity (homogeneity) of tobacco consumption among persons belonging to the same group [34]. The community-level ICC for persons belonging to the same community (and therefore the same state) is the sum of state-level and community-level variance divided by the total variance in the model [35]. The state-level ICC for persons belonging to the same state (but not necessarily from the same community) is the proportion of state-level variance out of the total variance.

Extant studies have shown that it is incorrect to undertake cross-group comparisons of odds ratios obtained from logistic regression models, even if they have a similar set of dependent and independent variables [36, 37]. Therefore, to overcome this limitation and facilitate comparisons of the risk of tobacco consumption across both rounds of IHDS, we estimated marginal predicted probabilities of combustible tobacco consumption (or smokeless tobacco consumption) for a particular independent variable, at the median values (margins) of other independent variables [37].

We checked for multicollinearity in the multivariable regression models and found that the mean-variance inflation factor (VIF) across all models was less than 2.85 in both rounds, implying the non-necessity of adjusting for multicollinearity in the regression models [38]. All statistical estimations in the study were performed using the STATA software, version 13.0 [39].

## Results

### Descriptive analysis

Table 1 represents the numeric (N) and percentage (%) population distribution by relevant socioeconomic and demographic characteristics in the cross-sectional and panel datasets during IHDS round-I and round-II, respectively. We found that about 67 and 79% of household members watched TV in 2004–05 and 2011–12. Nearly 48 and 28% of household members listened to the radio in 2004–05 and 2011–12. About 39 and 48% of household members read newspapers in 2004–05 and 2011–12. About 7% of the study population had more than 10 years of schooling in 2004–05 and 2011–12.

**Table 1:** Population distribution by explanatory characteristics in the cross-sectional and panel datasets during two IHDS rounds

Characteristics	IHDS round-I					IHDS round-II				
	Cross-sectional dataset		Panel dataset		Absolute difference	Cross-sectional dataset		Panel dataset		Absolute difference
	N	%	N	%	%	N	%	N	%	%
**Household members watch TV**										
No	10,126	30.6	5538	33.2	2.6	6398	18.8	3447	20.7	1.9
Yes	22,990	69.4	11,123	66.8	2.6	27,692	81.2	13,214	79.3	1.9
**Household members listen to radio**										
No	16,930	51.1	8724	52.4	1.3	24,172	70.9	11,910	71.5	0.6
Yes	16,186	48.9	7937	47.6	1.3	9918	29.1	4751	28.5	0.6
**Household members read newspaper**										
No	18,576	56.1	10,186	61.1	5.0	16,584	48.6	8620	51.7	3.1
Yes	14,540	43.9	6475	38.9	5.0	17,506	51.4	8041	48.3	3.1
**Age group of individual**										
Children and youth	2411	7.3	1004	6.0	1.3	2510	7.4	90	0.5	6.9
Adults	27,151	82.0	14,527	87.2	5.2	27,095	79.5	13,952	83.7	4.2
Elderly	3554	10.7	1130	6.8	3.9	4485	13.2	2619	15.7	2.5
**Gender of individual**										
Male	27,609	83.4	14,889	89.4	6.0	28,306	83.0	14,892	89.4	6.4
Female	5507	16.6	1772	10.6	6.0	5784	17.0	1769	10.6	6.4
**Level of education of individual**										
No formal schooling	13,555	40.9	6984	41.9	1.0	12,542	36.8	6881	41.3	4.5
1–5 years of schooling	6715	20.3	3581	21.5	1.2	6947	20.4	3738	22.4	2.0
6–10 years of schooling	9825	29.7	4935	29.6	0.1	10,959	32.1	4810	28.9	3.2
More than 10 years of schooling	3021	9.1	1161	7.0	2.1	3642	10.7	1232	7.4	3.3
**Current working status of individual**										
Not working	15,373	46.4	6787	40.7	5.7	7191	21.1	2705	16.2	4.9
Working	17,743	53.6	9874	59.3	5.7	26,899	78.9	13,956	83.8	4.9
**Current marital status of individual**										
Currently married	28,455	85.9	15,123	90.8	4.9	28,335	83.1	14,884	89.3	6.2
Currently not married	4661	14.1	1538	9.2	4.9	5755	16.9	1777	10.7	6.2
**Wealth quintile of household**										
Poorest	7460	22.5	4442	26.7	4.2	7463	21.9	4060	24.4	2.5
Poor	7147	21.6	3897	23.4	1.8	7933	23.3	4108	24.7	1.4
Medium	6958	21.0	3585	21.5	0.5	7399	21.7	3703	22.2	0.5
Rich	6547	19.8	2903	17.4	2.4	6158	18.1	2850	17.1	1.0
Richest	5004	15.1	1834	11.0	4.1	5137	15.1	1940	11.6	3.5
**Caste of household head**										
Other Backward Classes	12,811	38.7	6634	39.8	1.1	13,649	40.1	6576	39.5	0.6
Scheduled Castes	7521	22.7	4066	24.4	1.7	8005	23.5	4111	24.7	1.2
Scheduled Tribes	3983	12.0	1944	11.7	0.3	3921	11.5	1974	11.8	0.3
Others	8801	26.6	4017	24.1	2.5	8470	24.9	4000	24.0	0.9
**Religion of household head**										
Hindu	27,046	81.7	13,945	83.7	2.0	28,235	82.8	14,100	84.6	1.8
Muslim	3657	11.0	1740	10.4	0.6	3980	11.7	1754	10.5	1.2
Others	2413	7.3	976	5.9	1.4	1875	5.5	807	4.8	0.7
**Place of residence**										
Rural	24,641	74.4	13,223	79.4	5.0	24,795	72.7	12,904	77.5	4.8
Urban	8475	25.6	3438	20.6	5.0	9295	27.3	3757	22.5	4.8
**Country regions**										
Central	7663	23.1	4898	29.4	6.3	9615	28.2	4898	29.4	1.2
Northern	6522	19.7	3368	20.2	0.5	6925	20.3	3368	20.2	0.1
Southern	5683	17.2	2418	14.5	2.7	5566	16.3	2418	14.5	1.8
Western	4302	13.0	1931	11.6	1.4	4186	12.3	1931	11.6	0.7
Eastern	6572	19.8	3356	20.1	0.3	6206	18.2	3356	20.1	1.9
North-eastern	2374	7.2	690	4.1	3.1	1592	4.7	690	4.1	0.6
**Overall**	**33,116**	**100**	**16,661**	**100**	**0**	**34,090**	**100**	**16,661**	**100**	**0**

From the “absolute difference” column, we observe that the percentage population distribution by socioeconomic and demographic characteristics is similar across the cross-sectional and panel datasets in both rounds of IHDS, respectively. In round-I, the distribution of persons by gender and country regions differed by more than 6% between the cross-sectional and panel datasets. Similarly, population distribution by gender and current marital status differed by more than 6% during round-II.

### Bivariate analysis

Table 2 presents the bivariate association of relevant individual-level, socioeconomic and community-level variables with combustible and smokeless tobacco consumption during 2004–05 and 2011–12, respectively. A higher percentage of individuals who do not watch TV consumed combustible tobacco [61% in 2004–05 and 54% in 2011–12]. Nearly 62% and 53% of the study population who listened to radio indulged in combustible tobacco in 2004–05 and 2011–12, respectively. Similarly, a higher percentage of individuals who do not read newspapers consumed combustible tobacco [62% in 2004–05 and 53% in 2011–12]. A higher proportion of adults [62% in 2004–05 and 53% in 2011–12] consumed combustible tobacco, whereas a higher percentage of males consumed combustible tobacco [66% in 2004–05 and 58% in 2011–12]. Communities with a lower percentage of the non-educated population had a higher percentage of combustible tobacco consumption in 2004–05 but the relationship inversed in 2011–12. Similarly, a community with a lower percentage of poor individuals had a higher percentage of combustible tobacco consumption in 2004–05 and 2011–12. More rural residents consumed combustible tobacco [62% in 2004–05 and 54% in 2011–12]. Combustible tobacco consumption was highest in the northern region of India [83% in 2004–05 and 77% in 2011–12].

**Table 2:** Bivariate analysis showing the association of individual-level, community-level and relevant socioeconomic variables with combustible and smokeless tobacco use during IHDS round-I and round-II, respectively

Characteristics	IHDS round-I						IHDS round-II					
	Combustible tobacco			Smokeless tobacco			Combustible tobacco			Smokeless tobacco		
	Total	Yes	Chi^2^ test *p*-value	Total	Yes	Chi^2^ test *p*-value	Total	Yes	Chi^2^ test *p*-value	Total	Yes	Chi^2^ test *p*-value
	N	%		N	%		N	%		N	%	
**Household members watch TV**												
No	5538	61.1	0.765	5538	61.6	< 0.001	3447	53.6	0.298	3447	63.2	< 0.001
Yes	11,123	60.9		11,123	57.3		13,214	52.6		13,214	58.0	
**Household members listen to radio**												
No	8724	59.8	0.002	8724	57.2	< 0.001	11,910	52.6	0.463	11,910	58.4	0.003
Yes	7937	62.2		7937	60.5		4751	53.3		4751	60.9	
**Household members read newspaper**												
No	10,186	61.8	0.009	10,186	59.7	0.001	8620	53.2	0.256	8620	61.2	< 0.001
Yes	6475	59.7		6475	57.2		8041	52.4		8041	56.8	
**Age group of individual**												
Children and youth	1004	46.8	< 0.001	1004	73.1	< 0.001	90	31.1	< 0.001	90	83.3	< 0.001
Adults	14,527	62.2		14,527	57.5		13,952	53.0		13,952	59.0	
Elderly	1130	58.0		1130	61.7		2619	52.4		2619	58.7	
**Gender of individual**												
Male	14,889	66.3	< 0.001	14,889	55.4	< 0.001	14,892	57.5	< 0.001	14,892	55.7	< 0.001
Female	1772	16.3		1772	87.1		1769	13.1		1769	87.9	
**Level of education of individual**												
No formal schooling	6984	60.4	< 0.001	6984	60.2	< 0.001	6881	53.0	< 0.001	6881	61.3	< 0.001
1–5 years of schooling	3581	63.3		3581	59.6		3738	55.8		3738	58.2	
6–10 years of schooling	4935	61.7		4935	55.6		4810	52.4		4810	56.4	
More than 10 years of schooling	1161	53.8		1161	60.5		1232	44.2		1232	60.4	
**Current working status of individual**												
Not working	6787	57.6	< 0.001	6787	61.3	< 0.001	2705	38.0	< 0.001	2705	66.1	< 0.001
Working	9874	63.3		9874	57.0		13,956	55.7		13,956	57.7	
**Current marital status of individual**												
Currently married	15,123	62.4	< 0.001	15,123	57.7	< 0.001	14,884	54.1	< 0.001	14,884	58.0	< 0.001
Currently not married	1538	47.1		1538	69.4		1777	42.4		1777	68.0	
**Wealth quintile of household**												
Poorest	4442	54.6	< 0.001	4442	71.3	< 0.001	4060	49.0	< 0.001	4060	73.0	< 0.001
Poor	3897	62.6		3897	62.3		4108	52.4		4108	63.6	
Medium	3585	64.8		3585	53.1		3703	55.1		3703	54.7	
Rich	2903	64.2		2903	50.1		2850	55.3		2850	48.6	
Richest	1834	60.3		1834	45.5		1940	53.7		1940	44.2	
**Caste of household head**												
Other Backward Classes	6634	59.5	< 0.001	6634	62.1	< 0.001	6576	51.7	< 0.001	6576	62.6	< 0.001
Scheduled Castes	4066	65.2		4066	55.6		4111	57.8		4111	54.9	
Scheduled Tribes	1944	50.1		1944	72.5		1974	43.3		1974	68.7	
Others	4017	64.5		4017	49.6		4000	54.3		4000	52.8	
**Religion of household head**												
Hindu	13,945	60.4	< 0.001	13,945	59.3	< 0.001	14,100	52.4	< 0.001	14,100	60.3	< 0.001
Muslim	1740	69.5		1740	53.0		1754	60.8		1754	53.2	
Others	976	53.7		976	61.1		807	43.5		807	50.4	
**Percentage of individuals in community with no formal education**												
0 to 25%	4076	62.2	0.036	4076	52.6	< 0.001	4220	53.1	0.030	4220	52.9	< 0.001
25 to 50%	5549	61.4		5549	60.5		5602	53.4		5602	60.1	
50 to 75%	5224	59.4		5224	63.5		5160	51.3		5160	64.0	
75 to 100%	1812	61.4		1812	53.4		1679	54.9		1679	56.1	
**Percentage of individuals in community from poorest/poor wealth quintile**												
0 to 25%	5205	67.4	< 0.001	5205	44.5	< 0.001	5476	57.5	< 0.001	5476	44.8	< 0.001
25 to 50%	2553	64.4		2553	51.9		2637	55.0		2637	55.6	
50 to 75%	3195	57.5		3195	63.3		3237	51.5		3237	63.7	
75 to 100%	5708	55.6		5708	72.2		5311	47.8		5311	72.7	
**Percentage of individuals in community belonging to SC/ST caste**												
0 to 25%	6835	62.6	< 0.001	6835	56.1	< 0.001	6837	53.7	< 0.001	6837	58.1	< 0.001
25 to 50%	4443	59.2		4443	61.1		4337	52.6		4337	61.8	
50 to 75%	2663	62.7		2663	56.6		2860	54.8		2860	56.8	
75 to 100%	2720	58.2		2720	63.7		2627	48.8		2627	59.6	
**Place of residence**												
Rural	13,223	61.5	0.003	13,223	59.2	0.012	12,904	53.8	< 0.001	12,904	59.9	< 0.001
Urban	3438	58.8		3438	56.9		3757	49.5		3757	56.4	
**Country regions**												
Central	4898	58.6	< 0.001	4898	71.7	< 0.001	4898	52.8	< 0.001	4898	72.4	< 0.001
Northern	3368	83.2		3368	24.1		3368	76.8		3368	29.3	
Southern	2418	66.2		2418	38.3		2418	61.0		2418	41.1	
Western	1931	35.7		1931	76.4		1931	28.3		1931	75.5	
Eastern	3356	50.2		3356	75.3		3356	36.0		3356	74.5	
North-eastern	690	73.9		690	78.0		690	58.0		690	52.3	
**Overall**	**16,661**	**61.0**		**16,661**	**58.7**		**16,661**	**52.8**		**16,661**	**59.1**	

^(a)^Significance of the Chi-square (Chi^2^) test for association is shown using *p*-value

A higher percentage of individuals, who do not watch TV, consumed smokeless tobacco [62% in 2004–05 and 63% in 2011–12]. Similarly, a higher percentage of individuals who do not read newspapers consumed smokeless tobacco [60% in 2004–05 and 61% in 2011–12]. More children and youth consumed smokeless tobacco [73% in 2004–05 and 83% in 2011–12]. A higher percentage of females consumed smokeless tobacco [87% in 2004–05 and 88% in 2011–2012]. Communities with a higher proportion of poor individuals had a higher smokeless tobacco consumption. The prevalence of smokeless tobacco consumption was high among rural residents [59% in 2004–05 and 60% in 2011–12]. Smokeless tobacco consumption was highest in the western region of India [76% in 2004–05 and 76% in 2011–12].

### Multivariable analysis

The fixed-effect part of Tables 3 and 4 shows the multivariable association between combustible and smokeless tobacco consumption with mass media exposure using random-intercept logistic regression models during IHDS round-I and round-II, respectively. We found that watching TV [(OR:1.03; CI:0.92–1.15) in 2004–05 and (OR:0.99; CI:0.88–1.12) in 2011–12], listening radio [(OR: 0.99; CI:0.90–1.10) in 2004–05 and (OR:1.04; CI:0.94–1.15) in 2011–12] and reading newspaper [(OR:1.02; CI:0.91–1.15) in 2004–05 and (OR:0.96; CI:0.87–1.06) in 2011–12] did not have any significant effect on consumption of combustible tobacco. Similarly, watching TV [(OR:1.02; CI:0.91–1.15) in 2004–05) and (OR:1.11; CI:0.98–1.25) in 2011–12], listening radio [(OR:0.92; CI:0.83–1.02) in 2004–05 and (OR:0.91; CI:0.82–1.02) in 2011–12] and reading newspaper [(OR:1.05; CI:0.93–1.19) in 2004–05 and (OR:1.01; CI:0.91–1.12) in 2011–12] did not have any significant effect on consumption of smokeless tobacco.

**Table 3:** Multivariable association between tobacco use with mass media exposure and community-level and state-level effects from random intercept logit models during IHDS round-I

	IHDS round-I	
	Combustible tobacco^(d)^	Smokeless tobacco^(e)^
	OR (95% CI)	OR (95% CI)
***Fixed effect characteristics***		
**Household members watch TV**		
No	Ref.	Ref.
Yes	1.03 (0.92, 1.15)	1.02 (0.91, 1.15)
**Household members listen to radio**		
No	Ref.	Ref.
Yes	0.99 (0.90, 1.10)	0.92 (0.83, 1.02)
**Household members read newspaper**		
No	Ref.	Ref.
Yes	1.02 (0.91, 1.15)	1.05 (0.93, 1.19)
**Age group of individual**		
Children and youth	Ref.	Ref.
Adults	2.51* (2.10, 3.00)	0.40* (0.32, 0.48)
Elderly	2.24* (1.76, 2.85)	0.48* (0.37, 0.63)
**Gender of individual**		
Male	Ref.	Ref.
Female	0.059* (0.049, 0.070)	5.15* (4.25, 6.24)
**Percentage of individuals in community with no formal education**		
0 to 25%	Ref.	Ref.
25 to 50%	1.01 (0.83, 1.23)	1.16 (0.93, 1.45)
50 to 75%	0.92 (0.75, 1.14)	1.32* (1.04, 1.67)
75 to 100%	0.95 (0.72, 1.27)	0.82 (0.60, 1.12)
**Percentage of individuals in community from poorest/poor wealth quintile**		
0 to 25%	Ref.	Ref.
25 to 50%	0.90 (0.75, 1.08)	1.26* (1.03, 1.55)
50 to 75%	0.99 (0.80, 1.23)	1.12 (0.88, 1.42)
75 to 100%	0.91 (0.72, 1.15)	1.36* (1.05, 1.76)
**Percentage of individuals in community belonging to SC/ST caste**		
0 to 25%	Ref.	Ref.
25 to 50%	0.99 (0.78, 1.25)	0.91 (0.70, 1.17)
50 to 75%	0.81 (0.63, 1.04)	1.16 (0.88, 1.53)
75 to 100%	0.88 (0.68, 1.15)	1.17 (0.88, 1.57)
**Place of residence**		
Rural	Ref.	Ref.
Urban	0.89 (0.73, 1.07)	1.06 (0.85, 1.31)
**Country regions**		
Central	Ref.	Ref.
Northern	5.39* (1.92, 15.1)	0.11* (0.03, 0.39)
Southern	1.47 (0.48, 4.49)	0.13* (0.03, 0.48)
Western	0.27* (0.08, 0.92)	1.06 (0.25, 4.44)
Eastern	0.79 (0.25, 2.47)	3.39 (0.86, 13.4)
North-eastern	2.80 (0.47, 16.8)	1.94 (0.23, 16.7)
***Random effect parameters***		
**Level 3: State**		
Variance	0.634	0.925
Intraclass Correlation Coefficient (in %)	12.20	15.59
**Level 2: Community**		
Variance	1.276	1.717
Intraclass Correlation Coefficient (in %)	36.74	44.54
**Likelihood ratio test**	***	***
**No of states**	21	21
**No of communities**	2175	2175
**No of persons**	16,661	16,661

^(a)^*OR* Odds ratio, *CI* 95% Confidence Interval

^(b)^Ref. represents the reference category

^(c)^Statistical significance is denoted by asterisks where * indicates *p*-value< 0.05, *** indicates *p*-value< 0.0001

^(d)^Combustible tobacco use categorized into – No, Yes

^(e)^Smokeless tobacco use categorized into – No, Yes

^(f)^The results are adjusted for level of education, working status, marital status, household wealth quintile, caste, religion of household head

^(g)^Likelihood ratio test shows the significance of using a multilevel logistic model over a standard logistic model

**Table 4:** Multivariable association between tobacco use with mass media exposure and community-level and state-level effects from random intercept logit models during IHDS round-II

	IHDS round-II	
	Combustible tobacco^(d)^	Smokeless tobacco^(e)^
	OR (95% CI)	OR (95% CI)
***Fixed effect characteristics***		
**Household members watch TV**		
No	Ref.	Ref.
Yes	0.99 (0.88, 1.12)	1.11 (0.98, 1.25)
**Household members listen to radio**		
No	Ref.	Ref.
Yes	1.04 (0.94, 1.15)	0.91 (0.82, 1.02)
**Household members read newspaper**		
No	Ref.	Ref.
Yes	0.96 (0.87, 1.06)	1.01 (0.91, 1.12)
**Age group of individual**		
Children and youth	Ref.	Ref.
Adults	3.58* (2.06, 6.22)	0.28* (0.14, 0.54)
Elderly	3.75* (2.13, 6.59)	0.26* (0.13, 0.51)
**Gender of individual**		
Male	Ref.	Ref.
Female	0.088* (0.072, 0.11)	5.29* (4.32, 6.48)
**Percentage of individuals in community with no formal education**		
0 to 25%	Ref.	Ref.
25 to 50%	1.04 (0.88, 1.23)	0.85 (0.70, 1.02)
50 to 75%	0.93 (0.77, 1.11)	1.01 (0.83, 1.24)
75 to 100%	0.96 (0.75, 1.22)	0.91 (0.69, 1.19)
**Percentage of individuals in community from poorest/poor wealth quintile**		
0 to 25%	Ref.	Ref.
25 to 50%	0.91 (0.78, 1.07)	1.26* (1.06, 1.51)
50 to 75%	0.93 (0.78, 1.12)	0.99 (0.81, 1.21)
75 to 100%	0.78* (0.64, 0.96)	1.13 (0.90, 1.41)
**Percentage of individuals in community belonging to SC/ST caste**		
0 to 25%	Ref.	Ref.
25 to 50%	1.01 (0.83, 1.23)	1.10 (0.88, 1.37)
50 to 75%	0.91 (0.73, 1.12)	1.18 (0.93, 1.49)
75 to 100%	0.88 (0.70, 1.12)	1.20 (0.93, 1.56)
**Place of residence**		
Rural	Ref.	Ref.
Urban	0.80* (0.68, 0.94)	1.17 (0.98, 1.40)
**Country regions**		
Central	Ref.	Ref.
Northern	3.78* (1.35, 10.6)	0.17* (0.05, 0.48)
Southern	1.28 (0.42, 3.95)	0.19* (0.06, 0.59)
Western	0.34 (0.10, 1.14)	1.25 (0.36, 4.27)
Eastern	0.30* (0.09, 0.96)	2.12 (0.66, 6.82)
North-eastern	1.57 (0.25, 9.67)	0.43 (0.06, 2.69)
***Random effect parameters***		
**Level 3: State**		
Variance	0.667	0.677
Intraclass Correlation Coefficient (in %)	14.01	13.29
**Level 2: Community**		
Variance	0.802	1.125
Intraclass Correlation Coefficient (in %)	30.87	35.38
**Likelihood ratio test**	***	***
**No of states**	21	21
**No of communities**	2175	2175
**No of persons**	16,661	16,661

^(a)^*OR* Odds ratio, *CI* 95% Confidence Interval

^(b)^Ref. represents the reference category

^(c)^Statistical significance is denoted by asterisks where * indicates *p*-value< 0.05, *** indicates *p*-value< 0.0001

^(d)^Combustible tobacco use categorized into – No, Yes

^(e)^Smokeless tobacco use categorized into – No, Yes

^(f)^The results are adjusted for level of education, working status, marital status, household wealth quintile, caste, religion of household head

^(g)^Likelihood ratio test shows the significance of using a multilevel logistic model over a standard logistic model

The random-effect part of Tables 3 and 4 provides the group-level effects (community-level and state-level variance and ICC) from the random intercept logit models during round-I and round-II. During round-I, the high community-level ICC (37% for combustible and 45% for smokeless tobacco consumption) indicates that people from the same community of the same state have a greater or lower likelihood of consumption than people from other communities of the same state (implying high correlation). Further, the high state-level ICC (12 and 16%) indicate a high correlation of combustible and smokeless tobacco consumption among individuals belonging to the same state. Similar observations can be made for round-II, where community-level ICC (31 and 35%) is high for combustible and smokeless tobacco consumption of people belonging to the same community. Moreover, the high state-level ICC (14% for combustible and 13% for smokeless tobacco consumption) indicate a high correlation of tobacco consumption among individuals belonging to the same state.

### Predicted probabilities

Table 5 presents marginal predicted probabilities of combustible and smokeless tobacco use from random intercept logistic regression models calculated at the median value of relevant person-level, community-level and socioeconomic variables during IHDS round-I and round-II, respectively. The probability of combustible tobacco consumption declined among individuals who watched television [MPP: 0.835 to 0.732], listened radio [MPP: 0.833 to 0.740] and read newspaper [MPP: 0.837 to 0.724] from 2004-05 to 2011–12 respectively. However, the probability of smokeless tobacco consumption increased among individuals who watched television [MPP: 0.236 to 0.271], listened to radio [MPP: 0221 to 0.253] and read newspapers [MPP: 0.245 to 0.273] from 2004-05 to 2011–12 respectively.

**Table 5:** Marginal predicted probabilities of combustible and smokeless tobacco use from random intercept logistic regression models calculated at the median value of relevant person-level, community-level and socioeconomic variables during IHDS round-I and round-II, respectively

Characteristics	IHDS round-I		IHDS round-II	
	Combustible tobacco^(b)^	Smokeless tobacco^(c)^	Combustible tobacco^(b)^	Smokeless tobacco^(c)^
	MPP	MPP	MPP	MPP
**Household members watch TV**				
No	0.831	0.232	0.734	0.251
Yes	0.835	0.236	0.732	0.271
**Household members listen to radio**				
No	0.835	0.236	0.732	0.271
Yes	0.833	0.221	0.740	0.253
**Household members read newspaper**				
No	0.835	0.236	0.732	0.271
Yes	0.837	0.245	0.724	0.273
**Age group of individual**				
Children and youth	0.668	0.435	0.433	0.571
Adults	0.835	0.236	0.732	0.271
Elderly	0.818	0.270	0.741	0.257
**Gender of individual**				
Male	0.835	0.236	0.732	0.271
Female	0.230	0.614	0.194	0.663
**Level of education of individual**				
No formal schooling	0.849	0.215	0.748	0.265
1–5 years of schooling	0.835	0.236	0.732	0.271
6–10 years of schooling	0.777	0.251	0.652	0.323
More than 10 years of schooling	0.721	0.274	0.567	0.359
**Current working status of individual**				
Not working	0.835	0.224	0.706	0.235
Working	0.835	0.236	0.732	0.271
**Current marital status of individual**				
Currently married	0.835	0.236	0.732	0.271
Currently not married	0.800	0.255	0.724	0.290
**Wealth quintile of household**				
Poorest	0.824	0.247	0.767	0.302
Poor	0.835	0.236	0.744	0.287
Medium	0.805	0.267	0.732	0.271
Rich	0.801	0.234	0.737	0.230
Richest	0.761	0.208	0.718	0.203
**Caste of household head**				
Other Backward Classes	0.824	0.232	0.704	0.284
Scheduled Castes	0.835	0.236	0.732	0.271
Scheduled Tribes	0.806	0.243	0.725	0.263
Others	0.799	0.251	0.672	0.301
**Religion of household head**				
Hindu	0.835	0.236	0.732	0.271
Muslim	0.851	0.229	0.751	0.271
Others	0.775	0.243	0.657	0.298
**Percentage of individuals in community with no formal education**				
0 to 25%	0.833	0.210	0.725	0.305
25 to 50%	0.835	0.236	0.732	0.271
50 to 75%	0.821	0.260	0.710	0.307
75 to 100%	0.826	0.179	0.716	0.285
**Percentage of individuals in community from poorest/poor wealth quintile**				
0 to 25%	0.849	0.197	0.751	0.228
25 to 50%	0.835	0.236	0.732	0.271
50 to 75%	0.848	0.215	0.737	0.226
75 to 100%	0.836	0.250	0.701	0.250
**Percentage of individuals in community belonging to SC/ST caste**				
0 to 25%	0.862	0.210	0.751	0.240
25 to 50%	0.861	0.195	0.752	0.258
50 to 75%	0.835	0.236	0.732	0.271
75 to 100%	0.846	0.237	0.726	0.275
**Place of residence**				
Rural	0.835	0.236	0.732	0.271
Urban	0.818	0.246	0.687	0.303
**Country regions**				
Central	0.775	0.704	0.681	0.662
Northern	0.949	0.207	0.890	0.250
Southern	0.835	0.236	0.732	0.271
Western	0.481	0.716	0.421	0.710
Eastern	0.731	0.889	0.391	0.806
North-eastern	0.906	0.822	0.771	0.457

^(a)^MPP stands for marginal predicted probability

^(b)^Combustible tobacco use is categorized into - No, Yes

^(c)^Smokeless tobacco use is categorized into - No, Yes

## Discussion

Using two rounds of IHDS, this panel study examined the association of combustible and smokeless tobacco consumption with mass media exposure among the Indian population considering the extent of clustering and heterogeneous risk of tobacco consumption at the state and community levels. The results revealed no significant association between mass media exposure and combustible and smokeless tobacco consumption across the two rounds. While comparing both rounds using marginal predicted probability, this study further shows a minimal change in smoking behaviour and an increment in smokeless tobacco consumption from the short-term to the long-term period after COTPA act implementation. It was worth noting that, in the short-term and long-term phase after the COTPA act implementation, exposure to television, radio and newspaper was no longer associated with tobacco consumption. These findings are similar to a 2015 Indian study that showed how strategies like banning advertisements had efficiently nullified the association between mass media exposure and tobacco consumption [40]. However, the results of this study were also contradictory with another Indian study, using 2005–06 data for 15–49 aged women and 15–54 aged men [24]. This study highlighted the association of television and radio with a higher prevalence of tobacco chewing among men and newspaper reading with a lower likelihood of smokeless tobacco consumption among women [24].

Further, the current study observed the presence of clustering among individuals and a significant level of unobserved contextual risk of combustible and smokeless tobacco at the community and state levels. Community-level clustering was more pronounced as compared to the state-level in both rounds. Although, along with the nationally-implemented acts, India had witnessed different community-level initiatives (e.g. tobacco-free village) for tobacco control [41] and the state administration partnership helping various states win the tag of “smoke-free state”. Some studies contradict such association, providing evidence of increment in tobacco use in movies to promote such behaviour among youngsters at both state and community levels [42]. India has various entertainment sources across different communities and states and diverse cultures. The content shown in such entertainment sources might be the reason for promoting combustible and smokeless tobacco in India.

This study further revealed that education among individuals and the community had helped decrease combustible tobacco consumption. Besides, the smokeless tobacco consumption had increased from the short-term to the long-term phase of COTPA act implementation, and this result was consistent with a couple of studies [24, 43]. Smoking was higher among adults, and the elderly, whereas women were inclined towards smokeless tobacco consumption, and such results are consistent with an extant Indian study [10]. The high prevalence of smokeless tobacco consumption among Indian women occurred because it was culturally acceptable among some communities [42, 43] and was readily available due to its inexpensiveness. Further, the growing campaigns [12] and efforts of the government to air anti-tobacco television ads [44] adversely affect smoking behaviour across the country, making the tobacco industry more inclined towards the marketing of smokeless tobacco and introducing it as a quick replacement for combustible tobacco.

Exposure to radio has been a common means of communication and entertainment among people for many years, unlike, television which was seen as a newcomer and yet influential to every individual’s life [45]. Radio is a means of communication available in different languages and is readily accepted by individuals irrespective of their literacy status or age. Also, radio usage are common among some communities whose individual’s sit together and usually share their experience and behaviour. In such a situation, any pro- and anti-tobacco advertisements can influence many individuals in a community. Like radio, a newspaper is also a media type commonly seen among some communities, but more than this, it is an individual choice media which is common among the literate and the higher section of society. Although radio and newspaper exposure was not associated with tobacco consumption, a higher amount of community-level clustering in tobacco consumption among the Indian population may be explained by the effect of mass media on the communities. Besides, the variation in geographic level factors was also consistent with an Indian study [14].

One of the key strengths of this study is that rather than providing any impact assessment of the COTPA act, we have tried to examine the changes from the short term to the long-term period of COTPA act implementation on the combustible and smokeless tobacco consumption behaviour among Indian population using panel data. The study provided the opportunity to understand how the growing ban of tobacco advertisements on mass media after COTPA act implementation had reduced combustible tobacco consumption but paved the way for increment in smokeless tobacco marketing due to their inexpensive and readily available nature. The study also provided significant evidence that the risk of smoking and consuming smokeless tobacco varies significantly at the community and state levels. However, the study has its shortcomings too. Although past literature had brought forward the association between tobacco use and mass media exposure before and after the COTPA act implementation, the present study could not analyze such association due to the unavailability of information in IHDS data. Moreover, the study assumed that exposure to mass media involves involuntary exposure to advertisements promoted by commercial organizations through these media. However, to verify this assumption, one needs data on the media content type that an individual is exposed to, which was not possible in this study due to a lack of data. Primary studies considering the quality of content in the mass media can be conducted to address this limitation. Lastly, this study examines the correlation between tobacco consumption and mass media exposure, and the findings do not imply causality.

## Conclusion

The present study found a minimal change in the significant effect of mass media on combustible tobacco consumption among the Indian population after the COTPA act implementation. However, an increment of smokeless tobacco consumption during the two rounds, along with higher community-level clustering in tobacco consumption, had indicated the growing burden of smokeless tobacco behaviour. In terms of research implications, the findings show that mass media exposure cannot be considered as a strong predictor of combustible tobacco consumption in the Indian population. However, there is a need to view the content of media exposure as the type of content usually changes with the type of  medium. In terms of policy implications, there is a need for policy reforms to curb the significant effect of factors that promotes smokeless tobacco consumption in India, along with health warning labels on all types of tobacco to increase awareness in the individuals [46]. Moreover, clustering implies that such policies need to be implemented in specific high-risk communities from high-risk states, thereby reducing the time and cost required for implementation.

## Data Availability

The datasets used for this study are publicly available from the Inter-university Consortium for Political and Social Research (ICPSR) data repository [25, 26].

## Abbreviations

IHDS: India Human Development Survey
COPTA: Cigarettes and Other Tobacco Products Act
OR: Odds ratio
ICC: Intra-class Correlation Coefficient
OBC: Other Backward Classes
SC: Scheduled Castes
ST: Scheduled Tribes
CI: Confidence Interval
